# Spatial performance analysis in basketball with CART, random forest and extremely randomized trees

**DOI:** 10.1007/s10479-022-04784-3

**Published:** 2022-06-03

**Authors:** Paola Zuccolotto, Marco Sandri, Marica Manisera

**Affiliations:** BODaI-Lab, University of Brescia, Brescia, Italy

**Keywords:** Algorithmic modeling, Probability machines, Basketball analytics, Spatial performance, 62-xx, 62-07

## Abstract

This paper proposes tools for spatial performance analysis in basketball. In detail, we aim at representing maps of the court visualizing areas with different levels of scoring probability of the analysed player or team. To do that, we propose the adoption of algorithmic modeling techniques. Firstly, following previous studies, we examine CART, highlighting strengths and weaknesses. With respect to what done in the past, here we propose the use of polar coordinates, which are more consistent with the basketball court geometry. In order to overcome CART’s drawbacks while maintaining its points of force, we propose to resort to CART-based ensemble learning algorithms, namely to Random Forest and Extremely Randomized Trees, which are shown to be able to give excellent results in terms of interpretation and robustness. Finally, an index is defined in order to measure the map’s graphical goodness, which can be used—jointly with measures of the out-of-sample error—to tune the algorithm’s parameters. The functioning of the proposed approaches is shown by the analysis of real data of the NBA regular season 2020/2021.

## Introduction

The interest toward sports analytics has been hugely growing in the recent years, also thanks to the increased availability of a wide spectrum of data, coming from a lot of different sources, both traditional and technologically advanced. More and more, people involved with sports at different levels are recognizing the importance of extracting information from data, with a big variety of purposes such as, for example, describing strengths and weaknesses of athletes, players or teams, discovering the elements which mostly affect performance, identify the best game strategies or organize targeted training sessions. All these possible aims of the sports data analysis are relevant in the field of operations research, because they can be considered as issues needing a problem-solving approach in a decision-making framework (Csató, 2021; Wright, 2009, 2016).

In this paper we focus on basketball. In this context, data can be basic statistics from box scores, notational analysis data, the so-called play-by-play log, big data sets obtained by means of GPS sensors or other player tracking systems, cameras, platforms, wearable technologies and other data such as those coming from psychometric questionnaires, market analysis, etc.

As regards to statistical scientific literature, beyond Dean Oliver’s milestone book (Oliver, 2004), where we find the definition of concepts such as pace and possessions, offensive and defensive efficiency ratings, Four Factors (Kubatko et al., 2007), a wide set of studies have been produced with the aim to investigate complex problems thanks to a variety of statistical techniques, ranging from traditional models to data mining and machine learning. Contributions have been made in different fields, such as the study of scoring patterns and the measurement of players’ performance (Avugos et al., 2013; Cervone et al., 2016; Deshpande & Jensen, 2016; Engelman 2017; Erčulj & Štrumbelj 2015; Fearnhead & Taylor,^2011^; Franks et al., 2016; Gilovich et al., 1985; Gabel & Redner, 2012; Özmen, 2012; Page et al., 2013; Passos et al., 2016; Schwarz, 2012; Zuccolotto et al., 2018), the identification of features characterizing successful and unsuccessful teams (Garcá et al., 2013; Koh et al., 2011; 2012), the analysis of players’ profiles with reference to roles and way of playing (Alagappan, 2012; Bianchi et al., 2017), the description of the network of ball passing or the players’ pathways and trajectories (Ante et al., 2014; Bornn et al., 2017; Clemente et al., 2015; Fewell et al., 2012; Gudmundsson & Hortonrton, 2017; Lamas et al., 2011; Metulini et al., 2017a; b; 2018; Miller & Bornn, 2017; Passos et al., 2011; Piette et al., 2011; Shortridge et al., 2014; Wu & Bornn, 2018), the prediction of the outcome of games (Gupta, 2015; Lopez & Matthews, 2015; Manner, 2016; Ruiz & Perez-Cruz, 2015; Vračar et al., 2016; Yuan et al., 2015), the analysis of the positive or negative synergies that specific combinations of players may create on the court (Sandri et al., 2020b), the design of optimal game strategies (Nikolaidis, 2015; Skinner & Goldman, 2017; Zhang et al., 2013), as well as the effects of rules (Wright, 2014), scheduling (Wright, 2006) and referees assignment (Durán et al., 2021).

A set of possible applications of data science to basketball is presented in a recent book (Zuccolotto & Manisera, 2020), where the analyses are performed by means of the new R package BasketballAnalyzeR,^1^ specifically devoted to basketball analytics (Manisera et al., 2019; Sandri, 2020; Sandri et al. 2020a).

Performance analysis is a hot topic in sports analytics. In basketball, it can be defined with reference to both offensive and defensive abilities or limiting attention to specific aspects. The most commonly investigated perspective is shooting performance, which can be examined with different approaches, such as investigating its pattern along a whole season, during a game, in specific game situations or with reference to different areas of the court.

In this paper we deal with spatial shooting performance and we propose some innovative tools to analyze players’ performance along the court. To date, the main methods used by analysts to describe spatial performance are basic statistics such as shooting percentages computed in predetermined regions (squares or slices) of the court (for example, some functions are available in BasketballAnalyzeR to this aim). A new method has been proposed by Zuccolotto et al. (2021), where CART (Classification And Regression Trees, Breiman et al., 1984) split the court into rectangles that are optimal with respect to a given player (or team) shooting performance, instead of into uniform grids or predefined regular slices. The strength of that method is that each player (or team) has his own partition of the court, that is optimal with respect to his (its) specific characteristics. In this paper we start from that idea and develop it in different directions: firstly, we adapt it to work in a polar coordinate system, in order to obtain a partition more consistent with the court geometry. Secondly, we propose to replace trees with ensemble learning algorithms, to overcome the typical instability of CART partitions. In fact, it is well known that CART are not robust with respect to changes in the tuning parameters and in the training data. Tuning parameters are those that can be controlled during the estimation of the model: for example, in CART, usual tuning parameters are the complexity parameter and minimum number of observations that must exist in a node after splitting. We show that the results obtained with ensemble learning algorithms are much more robust, as they remain substantially the same under a variety of conditions with respect to the choices made for tuning parameters and in the presence of different observations.

To the best of our knowledge, the graphical tool we propose in this paper is new in the basketball analytics literature. It should not be confused with the commonly used heatmaps of the basketball court, where colors simply denote areas with different shooting intensity. Here we estimate the conditional probability function for the binary outcome describing the made or missed shot, so that the resulting map highlights the spatial performance in terms of the player’s (or team’s) scoring probability. So, the main novelty of this contribution lies in exploiting statistical learning algorithms to develop this innovative graphical tool in a way that is both interpretable and robust. From a methodological point of view, in spite of the basic robustness to the choice of tuning parameters, we propose an index aimed at selecting their optimal values in this specific context. The index is based on two elements, namely the graphical appeal of the map and the out-of-sample error in the prediction of the shot outcome.

All the proposed procedures are explained with reference to real data case studies. Specifically, we analyzed play-by-play logs of the 1080 games played by the NBA teams during the regular season 2020/2021 (due to the COVID-19 pandemic, the regular season began on December 22, 2020 and was reduced to 72 games for each team). In these data, also called event-log, each event occurred during a game is recorded along with all the relevant information (time, quarter, play length, players involved, shot coordinates, etc). The data set has been kindly made available by BigDataBall (www.bigdataball.com), a spin-off project powered by renowned NBAstuffer, which provides sports data scientists with high quality analysis tools and is a reliable source of validated and verified data.

The paper is organized as follows: In Sect. 2 we make some brief preliminary observations about the methodological support of our proposals, which are based on the use of algorithmic modeling techniques (Breiman, 2001b) for probability estimation (namely, in our context, the scoring probability). In Sect. 3 we show the court partitions obtained thanks to CART applied to polar coordinates and we also discuss the issue of the results’ robustness. Section 4 introduces the two ensemble learning algorithms we propose to adopt in order to overcome the drawbacks of the first approach, specifically addressing the issues of the additional information they are able to extract from the data and the stability of the interpretation they allow. In Sect. 5 we describe the index proposed in order to select the optimal value of tuning parameters. Section 6 draws some concluding remarks.

## Background

From a methodological point of view, this work deals with the estimation of the probability of an event conditional to the values assumed by a set of predictors. Formally, let *Y* be the dichotomous random variable associated to a shot, which assumes values 1 and 0 if the shot is made or missed, respectively. We estimate

1 $$\begin{aligned} Pr(Y=1 | X_1, X_2), \end{aligned}$$

where $X_1$ and $X_2$ denote the shot coordinates in the basketball court. This problem is identical to the regression estimation problem where we aim at determining a function of the predictors able to estimate the expected value of *Y* (conditional probability function),

$$\begin{aligned} E(Y | X_1, X_2) = f(X_1, X_2), \end{aligned}$$

because $$E(Y | X_1, X_2)=Pr(Y=1 | X_1, X_2)$$.

Among the possible solutions of this general probability estimation problem, we can opt for treating it as a non-parametric regression problem, a task for which many algorithmic modeling techniques are available. Malley et al. (2012) use the term “probability machine” to denote machine learning methods used to estimate the conditional probability function for a binary outcome. This issue had been mentioned already in the seminal book on CART (Breiman et al., 1984), where the trees used for the estimation of individual probabilities were termed “probability estimation trees” (PETs).

In general, statistical methods performing well on the non-parametric regression problem will also perform well on the probability estimation problem. In particular, if $f(X_1, X_2)$ can be estimated consistently by ${\hat{f}}_n(X_1, X_2)$, this property reflects on the probability estimation (Kruppa et al., 2014). More formally, a non-parametric regression function estimate is $L^2$-consistent if its mean square error converges to 0. In our problem with only 2 covariates, $L^2$-consistency can be written as:

$$\begin{aligned} \lim _{n \rightarrow \infty } E_{X_1, X_2} \left[ f(X_1, X_2) - {\hat{f}}_n(X_1, X_2) \right] ^2 = 0. \end{aligned}$$

Consistency has been proven to hold for many different machine learning approaches, including versions of Random Forest (Biau et al., 2008; Scornet et al., 2015), Quantile Regression Forests (Meinshausen & Ridgeway, 2006), and other algorithms such as k-nearest neighbors and bagged nearest neighbors (Kruppa et al., 2014). Furthermore, Biau (2012) showed that the convergence rate only depends on the number of variables which are relevant for the prediction model and not on how many noise variables are present. Interesting further details can be found in Biau and Devroye (2010).

These remarks give solid methodological foundations to our proposal of using algorithmic modeling techniques for spatial performance estimation. In addition, it is important to emphasize that the main part of probability machines are fully non-parametric, in the sense that they do not need any distributional assumption for the predictors, do not impose any restriction on the number of predictors and do not require a specified model as a starting point. From a computational point of view, implementing a probability machine does not involve any additional coding effort and no changes are needed to the basic algorithms adopted for regression (Malley et al., 2012).

## Partition of the court with classification trees

Decision trees are algorithms for regression or classification able to model complex relationships joining accuracy and interpretability. The most popular examples of decision trees are, beyond the already mentioned CART, the Iterative Dichotomiser 3 (ID3, Quinlan, 1986) and its extension C4.5 (Quinlan, 1993), Chi-square automatic interaction detection (CHAID, Kass, 1980), Conditional Inference Trees (Hothorn et al., 2006). A unified framework for presenting decision trees as greedy algorithms and describing the various splitting criteria and pruning methodologies can be found in Rokach and Maimon (2005).

In this section we briefly recall the procedure proposed by Zuccolotto et al. (2021) to partition the court into a set of rectangles, characterized by homogeneous shooting performance of a given player (or team), which is based on CART applied to a basketball court where shots are located using a cartesian coordinate system. Subsequently we illustrate our proposal of using shot coordinates expressed in a polar coordinate system and show the results obtained on real data.

In general, the CART algorithm obtains predictions for a (numerical or categorical) dependent variable *Y*, based on the values of a set of predictors $X_1, X_2, \ldots , X_p$. The trees are built by recursive binary partitions of the predictors’ space, defined with the aim of maximally reducing the heterogeneity of *Y* thanks to the split or, correspondingly, to achieve maximum homogeneity of *Y* within the partitions. Heterogeneity is usually measured with the variance if *Y* is numerical (in this case we are dealing with regression trees), or with impurity measures, such as the Gini impurity $I_G$ or the Shannon entropy *H*, if *Y* is categorical (classification trees). In detail, if *Y* has *J* classes, the heterogeneity within a node is measured as

$$\begin{aligned} I_G = 1- \sum _{c=1}^J f_c^2 \ \ \ \ \ \text{ or }\ \ \ \ \ H= - \sum _{c=1}^J f_c \log f_c \end{aligned}$$

where $f_c$ is the fraction of subjects belonging to class *c* in the node. Within each partition, a simple prediction model is fitted to data (*e.g.* the average or the mode according to whether we are growing a regression or a classification tree, respectively). When *Y* is categorical with a dichotomous outcome, CART can be considered probability machines, in the sense specified in Sect. 2.

In the proposal of Zuccolotto et al. (2021) the CART is grown with data given by all the shots attempted by a specific player (or team) in a sufficiently high number of games. The dependent variable *Y* is the binary categorical variable, indicating whether the attempted shot scored a basket ($Y=1$) or not ($Y=0$). The predictors are the two numerical variables $X_{width}$ and $X_{height}$, denoting the cartesian space coordinates in the court of the attempted shot; the node heterogeneity is measured with the Gini impurity $I_G$. In general, when the *p* predictors are numerical, the CART algorithm induces a partition of the *p*-dimensional predictor space into hyper-rectangles. In this case, being $p=2$, the bi-dimensional predictor space is the basketball court itself and the hyper-rectangles are actually rectangles that can simply be visualized in a court map. This allows us to obtain a very insightful graphical representation, able to carry different kind of information: the way the court is partitioned informs about where successful shots tend to be concentrated and extra information can be added through the rectangles’ color, which can denote shooting percentages or other game variables such as, for example, the average time in the match when the shots in a given rectangle are attempted. This may inform, for example, about the fact that a player shoots from a high-performance rectangle very late during the game, which can be an information of paramount importance for defining a defense strategy.

The first proposal of this paper is to adapt the described procedure based on CART to work with two different predictors, namely $X_{\rho }$ and $X_{\theta }$, obtained by transforming $X_{width}$ and $X_{height}$ into a polar coordinate system with the pole in the basket. In this setting, $X_{\rho }$ denotes the distance from the basket along the polar axis (radius) and $X_{\theta }$ the angle with respect to the court baseline. The rectangles into which the predictors space is partitioned by CART are then transformed back into a cartesian coordinate system, where their shape turns out to be represented by circular sectors or portions of annuli.

**Fig. 1:**
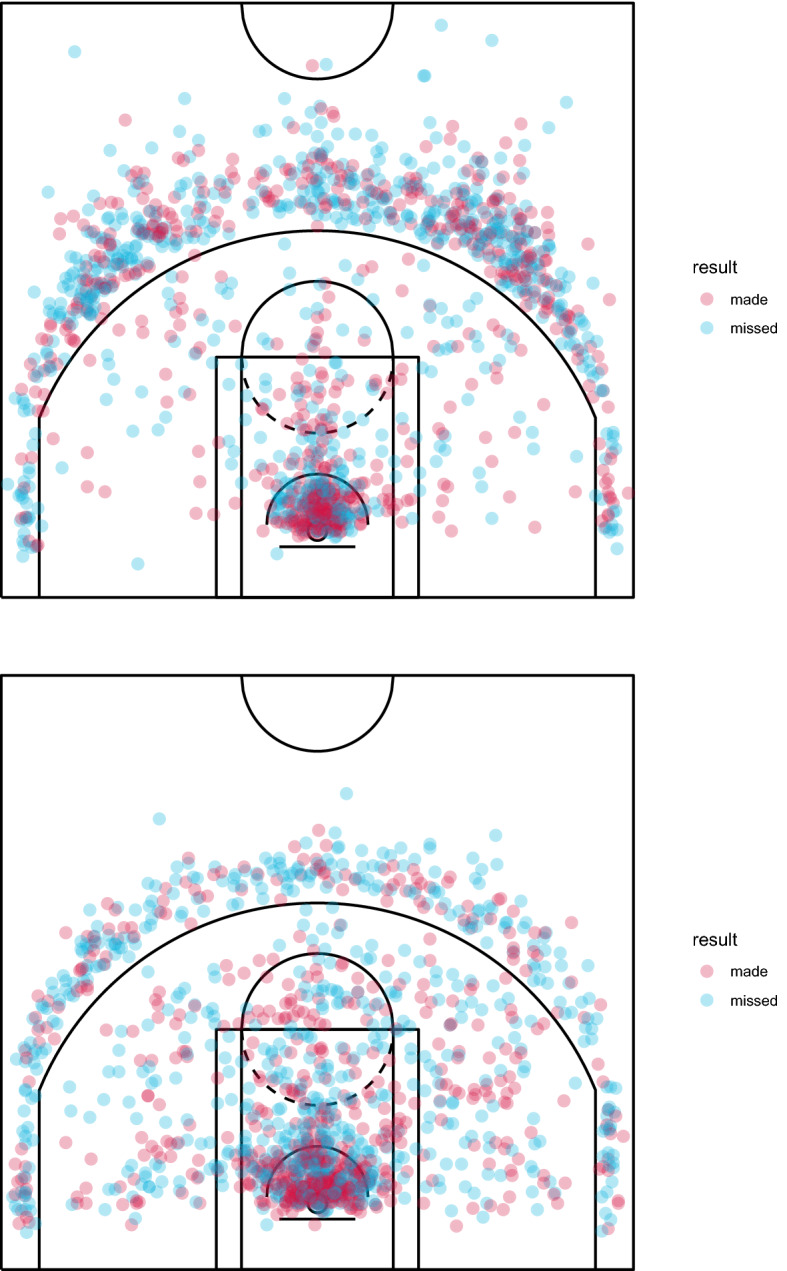
Shot charts with made and missed shots—Stephen Curry (top) and Bradley Beal (bottom), NBA regular season 2020/2021

We now present some examples of court partition based on a polar coordinate system. We analysed the players who ranked first and second as scoring leader of the NBA regular season 2020/2021, Stephen Curry (point guard, Golden State Warriors, 1365 shots from field) and Bradley Beal (shooting guard, Washington Wizards, 1382 shots from field), whose shot charts with made and missed shots are shown in Fig. 1.

**Fig. 2:**
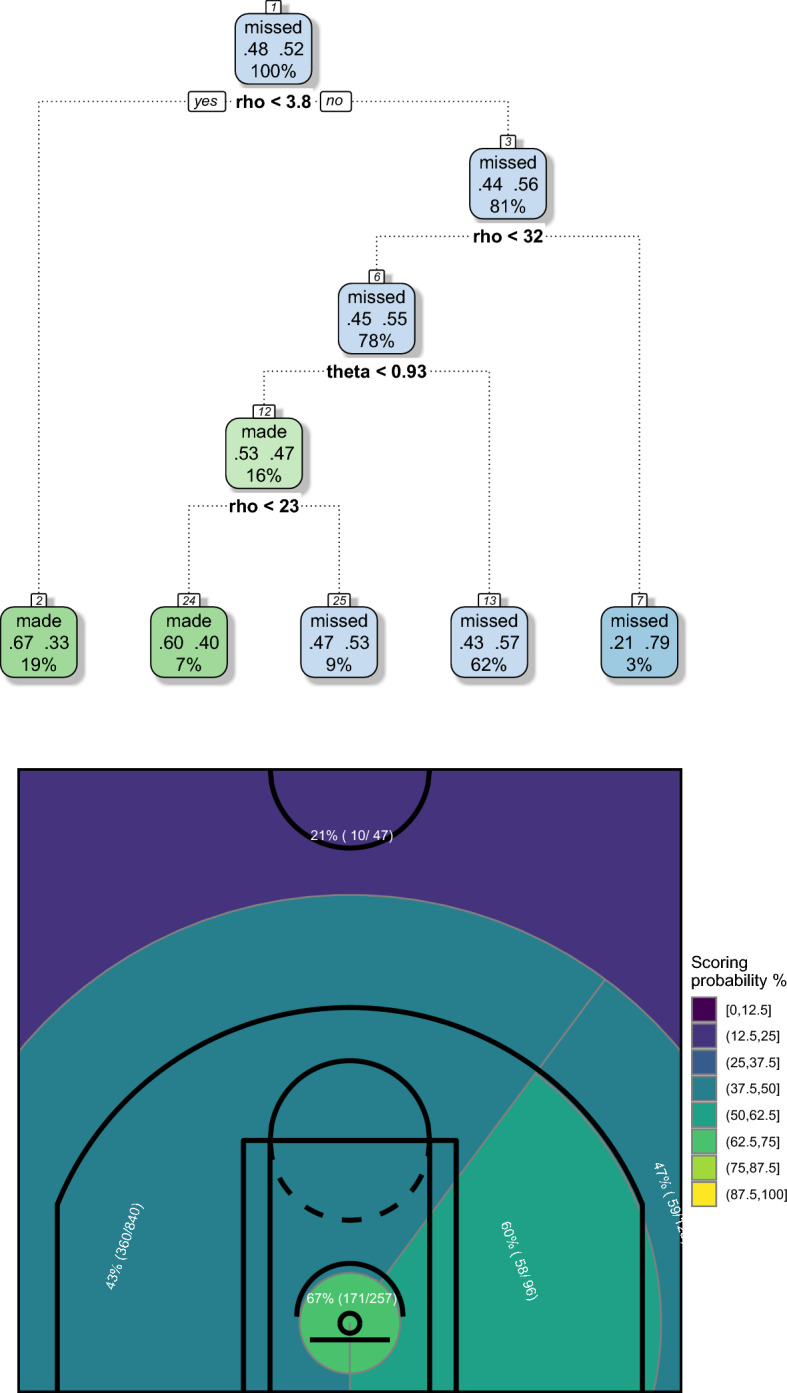
CART grown on polar coordinates (top) and corresponding court partition induced by CART (bottom)—Stephen Curry, NBA regular season 2020/2021

**Fig. 3:**
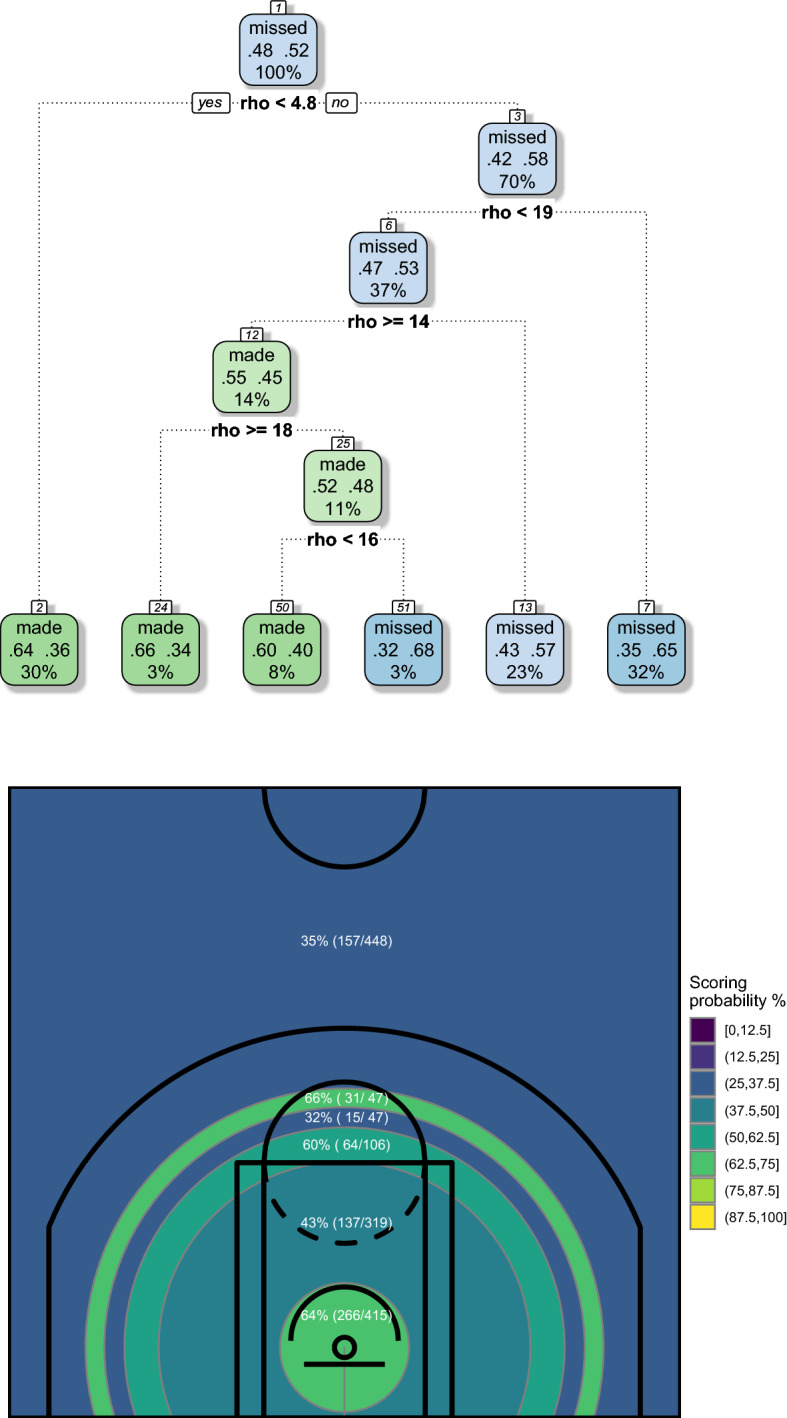
CART grown on polar coordinates (top) and corresponding court partition induced by CART (bottom)—Bradley Beal, NBA regular season 2020/2021

CART grown using polar coordinates and the corresponding court partitions are shown in Figs. 2 and 3. We adopted a pre-pruning strategy resorting to the usual control parameters available in the rpart R package. In detail, we have fixed a low complexity parameter (equal to 0.005), in order to detect also small performance differences, but a high minimum number of observations that must exist in a node in order for a split to be attempted (equal to the 10% of the total shots, so almost 140 for both players), in order to have reliable estimates of shooting percentages, based on a adequately high number of shots.

The top panels of Figs. 2 and 3 show the tree structures grown by the greedy CART algorithm. The nodes are labeled “made” or “missed” according to a majority rule and are correspondingly colored of green or blue, respectively. The higher the frequency of made or missed shots—and hence the lower the node misclassification error—the more intense the coloring. Focusing attention on the leaf nodes, for both Curry and Beal the frequency of made shots never exceeds 66–67%. Its lower value is 21%, in Curry’s map. The range of estimated probabilities is rather narrow, and this is another drawback that will be overcome by the ensemble learning algorithms as proposed in the next Section.

The bottom panels of Figs. 2 and 3 show the obtained court partitions, that are consistent with the two players’ characteristics.

In detail, Curry is well known to perform exceptionally well in shots from high distance, as clearly shown by his court partition (Fig. 2, bottom panel), where a great area with a scoring percentage of 43%, extended up to far behind the 3-point line, covers a big part of the court. However, we suspect that this big area might be further decomposed and some more specific spots could be identified within it. Another important result shown by the obtained map concerns the higher ability exhibited in shots from his right-hand side, a feature already detected in a previous study, based on data of the 2017/2018 regular season (Zuccolotto et al., 2021).

Beal enjoys a less established reputation than Curry, but experts claim that in the season 2020/2021 he has proven to be a mature player with his own characteristics, namely he has a not-exceptional ability in 3-point shots, but is able to be dangerous from inside the arc with a wide offensive repertory, which allows him to shoot from both the low and the middle distance. These features are confirmed by his court partition (Fig. 3, bottom panel). However, also in this case we feel that more information could be contained in the data, specifically it is a little bit surprising that not even a small difference between left or right emerges from the map.

**Fig. 4:**
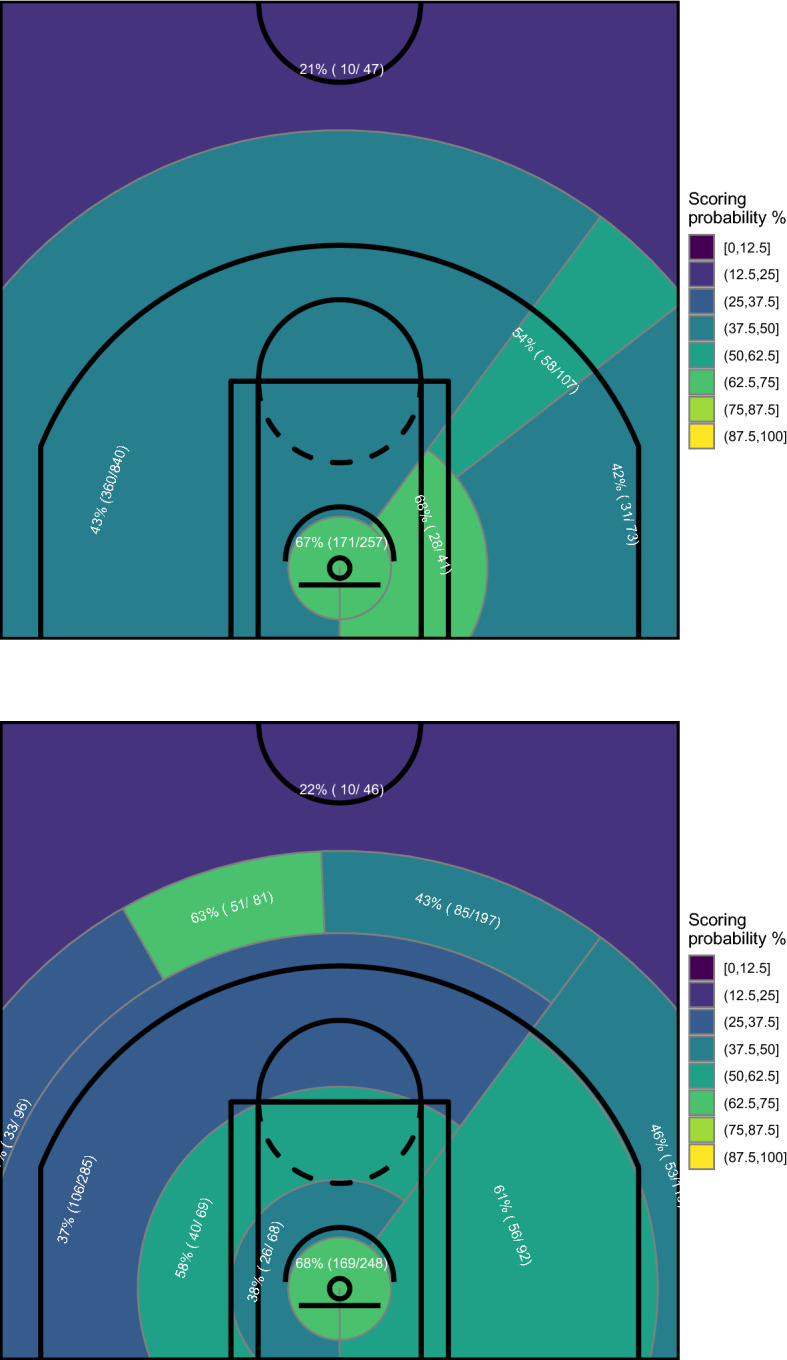
Court partitions induced by CART grown with minimum number of observations that must exist in a node in order for a split to be attempted set to 9% (top) and using a data set where a randomly selected 5% of shots was removed (bottom)—Stephen Curry, NBA regular season 2020/2021

In addition to the belief that more evidences could be extracted from the data at hand, this graphical tool suffers from some drawbacks, essentially related to three issues: (1) a certain amount of subjectivity in the pre-pruning criteria, (2) the well-known instability of CART and (3) the presence, in certain cases, of small areas difficult to interpret, whose definition is due to the rigid tree structure implied by the CART mechanism (for instance, see the blue thin area between the two green ones in Beal’s map). With respect to issues (1) and (2), we show in Fig. 4 the results obtained for Stephen Curry with a little modification of, respectively, the minimum number of observations that must exist in a node in order for a split to be attempted (set equal to the 9% of the total shots instead of 10%) and the data used to grow the tree (a randomly selected 5% of shots was removed from the data set).

The two court partitions shown in Fig. 4 raise an important point about the robustness of the graphical tool based on CART and also confirm the above mentioned suspect that the map of Fig. 2 may hidden some important information, like, for example, Curry’s particularly high ability in 3-point shots from middle-right (indicated by the green area in the bottom panel of Fig. 4). Similar conclusions can be reached with Beal’s data. Such appreciably different results as a consequence of little modifications in the data or in the parameters’ tuning are the main reason for the CART algorithm has lost some of its popularity in favor of ensemble learning algorithms (Friedman & Popescu, 2008), which overcome its main shortcomings. In the next section we propose the use of such methods in order to build more robust graphical representations of the players’ spatial performance.

## Spatial performance analysis with Random Forest and Extremely Randomized Trees

The most popular ensemble learning algorithms use CART as base learner: Random Forest (RF, Breiman, 2001a), Gradient Boosting Machine (GBM, Friedman, 2001), Extreme Gradient Boosting (XGBoost, Chen & Guestrin, 2016), Extremely Randomized Trees (ExtraTrees, Geurts et al., 2006). Since the method proposed in Sect. 3 is based on CART, a natural way to develop it in order to overcome its drawbacks is to refer to ensemble learning algorithms which adopt CART as base learner. In this paper we propose the use of RF and ExtraTrees, that are probably the most popular examples of CART-based ensemble learning algorithms built by injecting some kind of randomization into the tree growing process (see also Ali & Pazzani 1996; Amit & Geman, 1997; Cutler & Zhao, 2001; Ho, 1998).

In detail, RF grows multiple CARTs by selecting at random a subsample of data for each tree (bagging) and, in the end, merges them together to get a more accurate and stable prediction. At each node split, the search for the most effective feature in reducing heterogeneity of the target variable is done among a random subset of predictors, instead of the whole set. This introduces additional randomness to the procedure, which has been shown to improve model accuracy. The basic idea is that perturbations in the algorithm responsible for the tree growing help the mechanism to neutralize the variability of results (shown, for example, in Fig. 4), thanks to the fact that the final prediction is given by an average of those supplied by single trees. One further step of randomization yields ExtraTrees, that are still an ensemble of individual trees, with two main differences with respect to RF. Firstly, each tree is grown using the complete data set rather than a subsample (*i.e.*, without bagging). Secondly, in the node splitting a random cut-point selection is implemented by choosing, for each predictor among those randomly selected to split the node, one or more threshold values from a uniform distribution within the predictor’s empirical range. Among all these randomly generated splits, the one allowing the highest heterogeneity reduction in the target variable is then chosen to split the node.

With reference to the consistency property mentioned in Sect. 2, while it has been proven to hold for RF, to our best knowledge, an explicit proof has not been given for ExtraTrees. In Geurts et al. (2006), the Authors claim that from a purely theoretical point of view, one could ensure consistency of ExtraTrees under some conditions since, in this respect, this algorithm is not different from other tree-based methods and the proofs of consistency given in Breiman et al. (1984) still hold.

We point out that in our application we have only two predictors, which strongly limits the perturbation of the CART growing mechanism induced by the random selection of the predictors for the node splitting. For this reason, we believe that the additional randomization implemented by ExtraTrees can improve the final result with respect to RF.

**Fig. 5:**
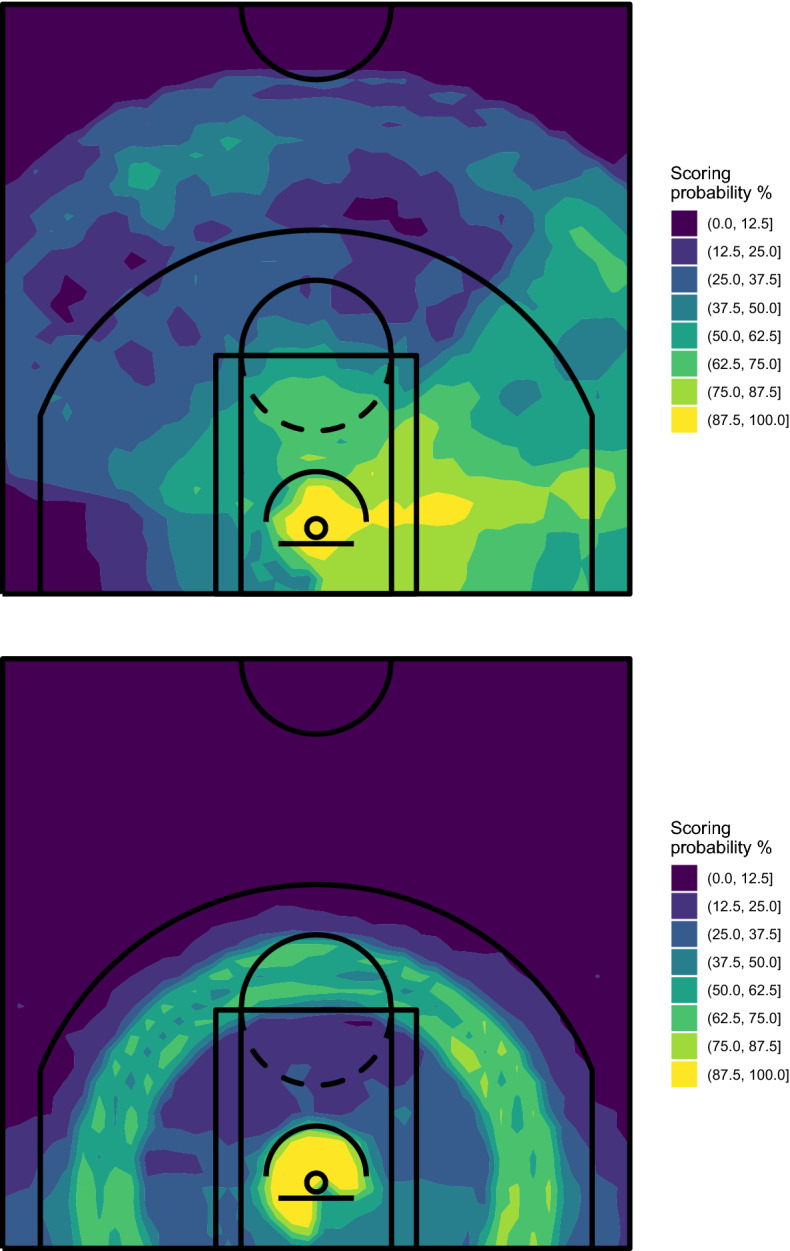
Spatial performance court maps obtained with RF—Stephen Curry (top) and Bradley Beal (bottom), NBA regular season 2020/2021

**Fig. 6:**
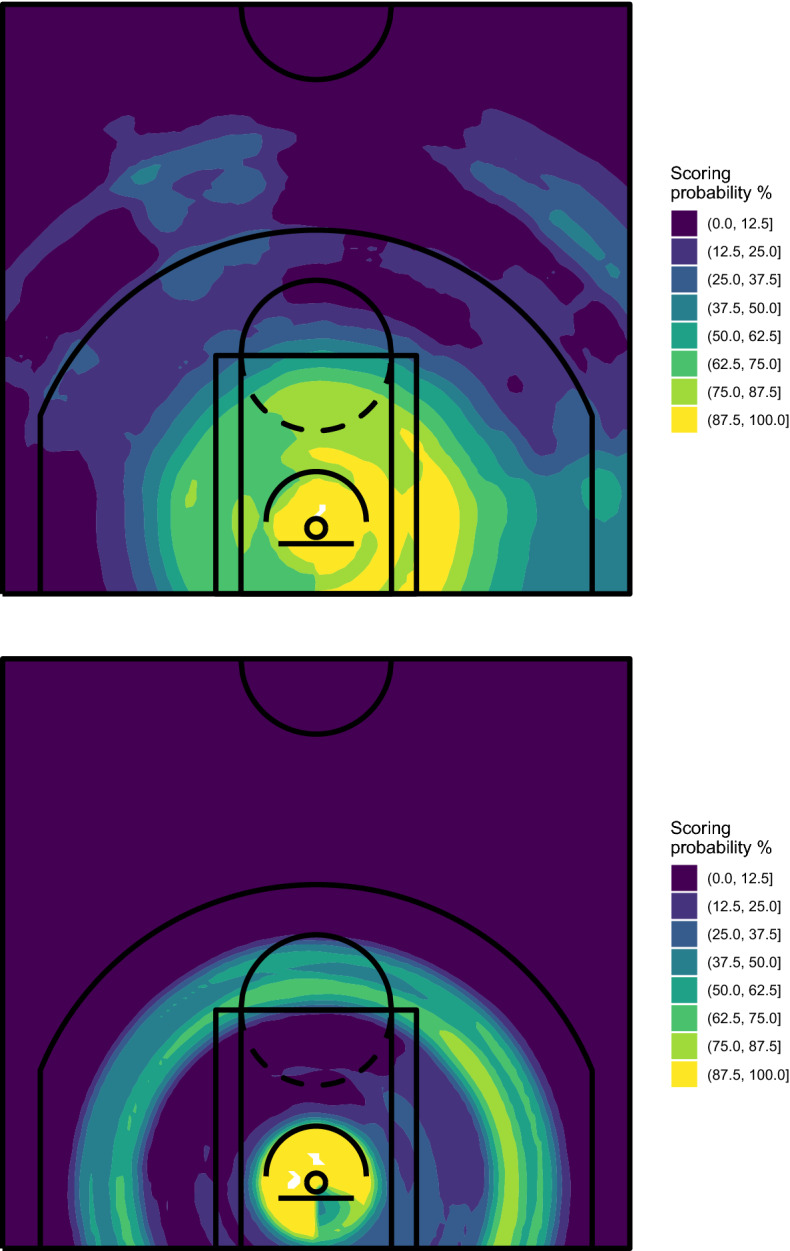
Spatial performance court maps obtained with ExtraTrees—Stephen Curry (top) and Bradley Beal (bottom), NBA regular season 2020/2021

In our proposal, the court map visualizing the players’ shooting performance is obtained by training the algorithm on the available data and then using it to predict the scoring probability on all the spots of a $100 \times 100$ grid built on the court. Figures 5 and 6 show the spatial performance court maps obtained with RF and ExtraTrees, respectively.

RF was grown with 5000 trees and using the default parameters of the randomForest R package, except for the minimum size of terminal nodes (which has been set to 200) and the number of predictors randomly selected for splitting the node (set to 2). However, the substantial information carried by the resulting map is not much affected by these choices, as we will show later. ExtraTrees was grown using the extraTrees R package, with 5000 trees, 2 randomly selected cut-points for each predictor at each node split and the remaining parameters equal to those used for RF. We have verified that also in these cases, the tuning parameters do not affect much the final result from a graphical point of view.

The maps shown in Figs. 5 and 6 substantially confirm the evidence already highlighted by the court partitions obtained with CART, but the range of estimated probabilities is appreciably larger, which denotes a higher ability of these algorithms in recognizing areas characterized by specific values of the scoring probability. Moreover, additional information is extracted from data. In detail, we find out that Curry’s shots from behind the 3-point line tend to be more dangerous from two specific spots, on the left and on the middle-right. For what concerns Beal, we discover a little preference for shots from his left.

**Fig. 7:**
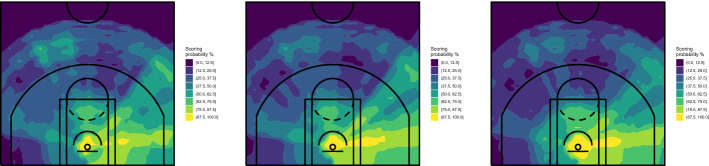
Spatial performance court maps obtained with RF, with minimum size of terminal nodes set to 150 (left), number of predictors randomly selected for splitting the node set to 1 (middle) and using a data set where a randomly selected 5% of shots was removed (right)—Stephen Curry, NBA regular season 2020/2021

**Fig. 8:**
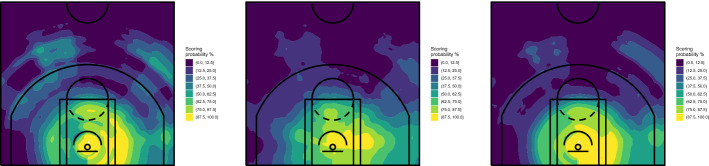
Spatial performance court maps obtained with ExtraTrees, with minimum size of terminal nodes set to 150 (left), number of predictors randomly selected for splitting the node set to 1 (middle) and using a data set where a randomly selected 5% of shots was removed (right)—Stephen Curry, NBA regular season 2020/2021

In addition to being able to extract hidden information, the ensemble learning algorithms provide more robust solutions than CART. We repeated the analyses using a different set of conditions. Specifically, (1) we reduced from 200 to 150 the minimum size of terminal nodes, (2) we reduced from 2 to 1 the number of predictors randomly selected for node splitting, and (3) we removed a 5% of shots from the training set ( i.e., we used the same data set used to grow the CART of Fig. 4, bottom panel). The results obtained for Stephen Curry are shown in Figs. 7 and 8, for RF and ExtraTrees, respectively.

Despite some obvious differences as a consequence of the parameters’ modification, the obtained maps do not show the degree of instability which characterized CART. The differences between the maps cover unessential details, while the substantial information is clearly visible in all cases. Similar conclusions can be reached with Beal’s data.

**Fig. 9:**
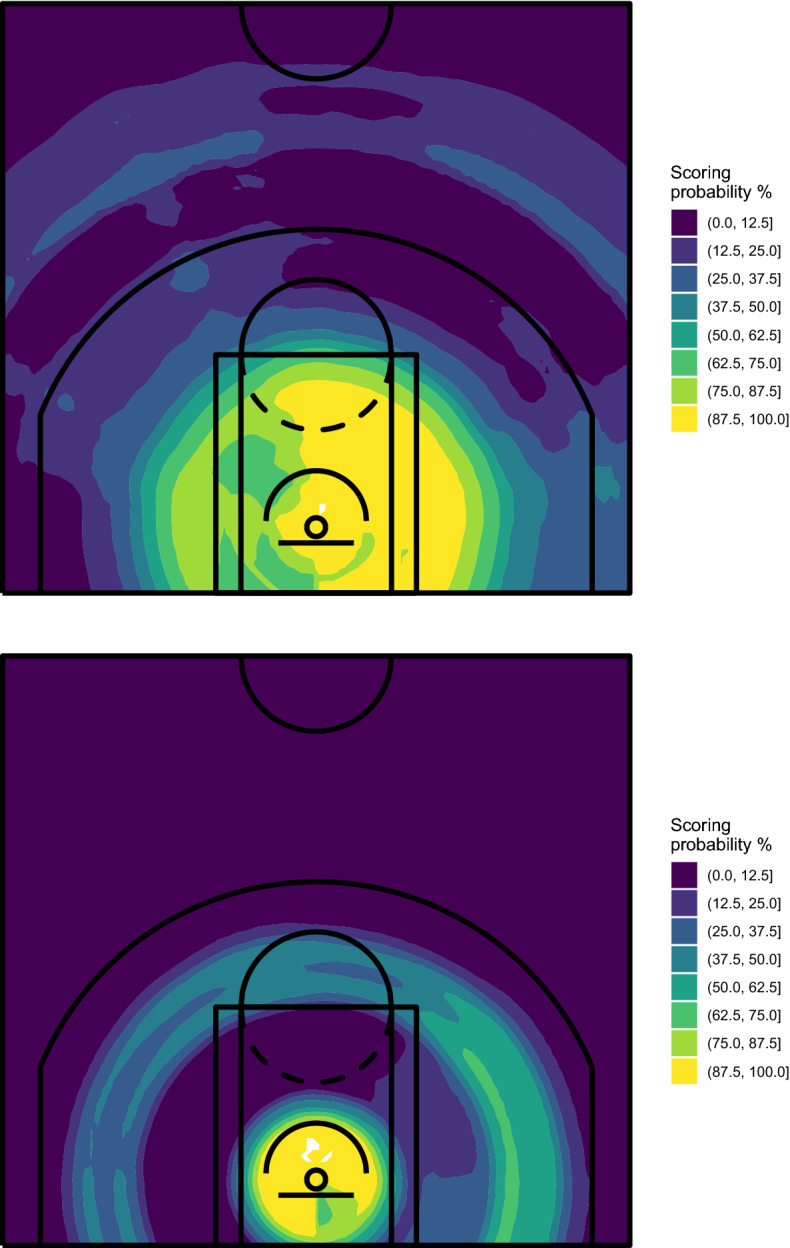
Spatial performance court maps obtained with ExtraTrees using a data set where a randomly selected 20% of shots was removed—Stephen Curry (top) and Bradley Beal (bottom), NBA regular season 2020/2021

We also explored more deeply the issue of the algorithm’s performance on different data, that is probably the most important source of instability of CART. In the bottom panel of Fig. 4 we showed the strong impact of a very little modification (only 5% of data removed). As demonstrated by the right panel of Figs. 7 and 8, the results obtained on the same data with RF and ExtraTrees are instead very similar to those on the whole data set. So, we tried a more severe action and removed a randomly selected 20% of shots. The results obtained with ExtraTrees for Curry and Beal are shown in Fig. 9. Also in this case, the map is still able to convey the same substantial evidence found in the whole data set. Similar conclusions can be reached with RF, although ExtraTrees exhibited slightly more stable results.

In order to evaluate the out-of-sample error of the obtained maps, we computed the 10-fold crossvalidated AUC. The obtained estimated values are rather low (around 0.6) and cannot be improved by acting on the tuning parameters, as we will show below. However, this poor performance from a predictive point of view only confirms that a shot made or missed is a very difficult event to predict, especially if we consider that, usually, there are wide areas in the map with an estimated scoring probability around 50%. As a matter of fact, we are not focused on prediction, but into performance evaluation: areas with scoring probability around 50% tell us that, there, the player’s performance has maximum variability, which is an important information itself. The fact that we are not able to efficiently predict whether his shots will be made or missed is a natural consequence, but we are just satisfied to know that the probability to score the basket is 50%.

**Fig. 10:**
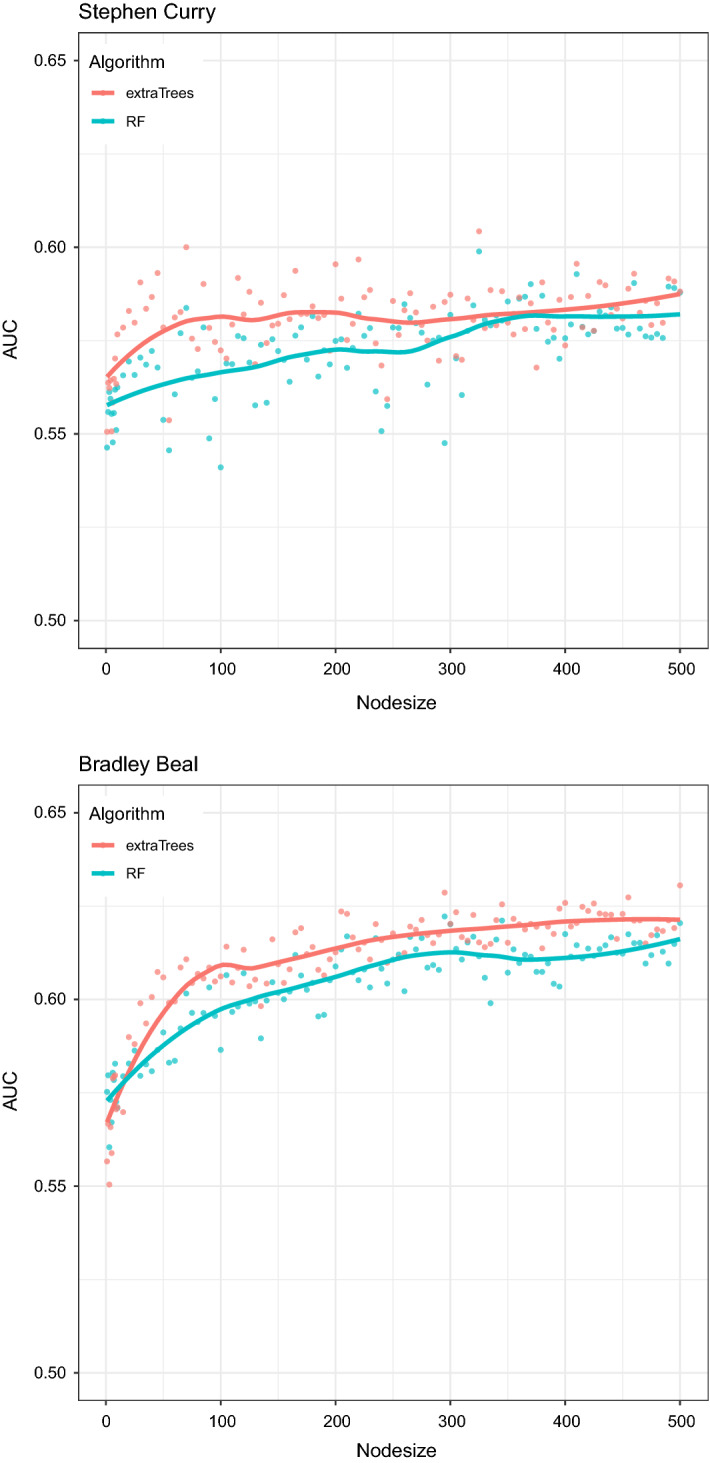
Out-of-sample AUC versus terminal node size in the RF and ExtraTrees algorithms—Stephen Curry (top) and Bradley Beal (bottom), NBA regular season 2020/2021

Another point is the choice of the tuning parameters. Although the algorithms appear robust with respect to this issue, we might as well ask for a formal tuning algorithm. In this respect, inspecting the out-of-sample error does not give definitive suggestions about the best choices, as we anticipated above, as the AUC values do not change much as a function of the tuning parameters. For example, computing the 10-fold crossvalidated out-of-sample AUC as a function of the terminal node size in both the RF and ExtraTrees algorithms, we obtain the patterns shown in Fig. 10. We clearly notice that—from the point of view of the predictive ability—the parameter corresponding to the minimum size of terminal nodes does not affect much the out-of-sample error, which exhibits an almost constant pattern. So, from this point of view, the node size can be set almost indifferently, provided that it is higher than 80/100 (for lower values, the low value of AUC is an indication of overfitting). Another important remark is that the out-of-sample error is almost the same for RF and ExtraTrees, with just a very slight superiority of the latter algorithm.

Summarizing, the evaluation of the prediction ability by means of an out-of-sample error measure:

- is not our primary concern,
- does not help us in parameters tuning,
- does not give an ultimate suggestion about which is the best algorithm between RF and ExtraTrees,
- however, it has necessarily to be performed in order to rapidly check that the map is not over- or underfitting the data.

We will return to the issues of the tuning algorithm and the choice of the optimal method between RF and ExtraTrees in the next section, where we will define an index able to assess the graphical goodness of the map.

## Measuring the graphical goodness of the spatial performance maps

In the previous section, we evaluated spatial performance maps by referring to their out-of-sample error, *i.e.* their ability to give good predictions of the outcome. To do that, we resorted to 10-fold crossvalidated out-of-sample AUC, a very commonly used method when the outcome is a categorical dichotomous variable. Of course, this is a fundamental feature, as an acceptable generalization error allows us to consider the map as a good representation of the player’s (team’s) scoring skills. At the same time, as we have already highlighted, this is not fundamental in our context, where the real aim is not prediction but performance evaluation.

In addition, in the previous section we showed that there are a lot of maps, obtained with different choices about the tuning parameters, having approximately the same out-of-sample error. The reader should have clearly perceived that maps obtained with different choices in the tuning parameters were all able to carry substantially the same information, but some were more “graphically appealing”.

The same happens when we have definitely to choose between using RF or ExtraTrees: out of what we showed in the previous Section, ExtraTrees seems to provide a less noisy graphical representation and a slightly higher robustness. Apart from the substantial equality of the out-of-sample error, we feel that the court maps obtained with ExtraTrees are more appealing from a merely graphical point of view. It’s worth pointing out that, in addition to Curry and Beal, we performed the same analysis for all the NBA players who attempted at least 700 shots in the 2020/2021 regular season (72 players). In almost all cases, ExtraTrees gave the same results of the case studies presented in this Section, from the point of view of out-of-sample error, ease of interpretation, graphical appeal, lack of noise in the map.

Then, we need a criterion to measure the goodness of the maps, with respect to the purpose for which they are built, that is an immediate and easy graphical interpretation of spatial performance. Following these remarks, in this section we give a formal definition of which features determine a map with a good graphical appeal, able to reach its goal of being a data visualization tool for spatial performance analysis. The definition of these characteristics will allow us to build an index able to measure the map’s goodness from this point of view, thus avoiding the assessment “by eye” we implicitly proposed in the previous analyses. The final aim is to have a tool allowing us both to tune parameters and to decide the best algorithm for our purposes.

Before we proceed, it is worth noting that the out-of-sample error and the graphical appeal are intimately connected with each other but, at the same time, they are not completely overlapped and assess the map from different perspectives. In fact, in presence of overfitting, it is very likely that the map will be undecipherable, but the same cannot be said in presence of underfitting. In addition, from a genuinely statistical point of view, the two assessments are made with two profoundly different approaches. The out-of-sample error is evaluated by checking the predictive ability on the available data, that is for the shots that have been effectively taken and, mainly, for the areas where these shots are more dense. The graphical appeal, instead, evaluates the appearance of the whole map, that is, in all the points of the grid where the estimated scoring probabilities are computed thanks to the algorithm. For the main part of these grid points we do not have a “ground truth” to which predictions can be compared and there are wide areas where the out-of-sample error cannot be verified, because no shots have been taken from there. These remarks should make clear the reason for we propose to assess the map from the two points of view and, definitely, to choose the “best” map by balancing these two aspects.

### Feature 1: Low spatial variability in the neighborhoods of points

The first feature we require to a good map is that the scoring probabilities estimated in all the *n* spots of the court,^2^${\hat{y}}_i$ ($=1,2, \ldots n$), are as spatially homogeneous as possible. Roughly speaking, we require that high/low/medium values of the estimated scoring probabilities tend to be close to each other. In practice, from a graphical point of view, we aim at having easily recognizable areas of different colors, as large as possible, thus avoiding a map which is undecipherable due to the presence of many different colors close each other. In order to measure this feature we propose to use an index based on the standard deviation of the values ${\hat{y}}_i$, computed in the neighborhoods of points:

2 $$\begin{aligned} \sigma _N = \sqrt{\frac{1}{n} \sum _{i=1}^n \sigma ^2_{N_i}} \end{aligned}$$

where $\sigma _{N_i}$ is the standard deviation of the scoring probabilities estimates of the points adjacent in space to the *i*th grid point. In general, we require low values of $\sigma _N$. Of course, a low value of $\sigma _N$, in itself, does not immediately guarantee a good map, because the different colors—beyond being homogeneous in the map—should also be present with the correct frequency distribution, in the sense that will be clarified in the following Feature 2.

### Feature 2: Non-uniform empirical distribution of the scoring probabilities

The probability distribution of the scoring probabilities is unknown and depends on the player. So, we do not have an assumption to which the empirical distribution of ${\hat{y}}_i$ can be compared. Nonetheless, we can broadly outline the following remarks.

- Shots are usually taken from a limited area within the court. Shots taken from a distance higher than 30 feet from the basket are uncommon and almost always result in a missed shot. Very roughly, this means that we can expect non-zero scoring probabilities for little more than half of the total area of the court.
- In the area from which shots are usually taken, even the best player misses some shots, if only for the defensive pressure of the opponent. So, we can expect very few scoring probabilities close to 1.

Following these considerations, we assume that the scoring probabilities estimated on the whole grid, ${\hat{y}}_i$, should have a strongly right-skewed distribution. We still do not know which specific distribution, but we know that it should be as far as possible from a Uniform distribution. A map with uniformly distributed estimated scoring probabilities, although being graphically appealing because all colors are equally represented, is not adequate to our purpose. We measure this characteristic by means of the following index, based on the Kolmogorov-Smirnov statistic for a cumulative distribution function:

3 $$\begin{aligned} H = \sup _y |{\hat{F}}(y) - F_U(y) | \end{aligned}$$

In formula (3), ${\hat{F}}(y)$ is the empirical distribution function of the estimated scoring probabilities of a given map,

$$\begin{aligned} {\hat{F}}(y) = \frac{1}{n}\sum _{i=1}^n I_{(-\infty ,y]}({\hat{y}}_i), \end{aligned}$$

where $I_{(-\infty ,x]}({\hat{y}}_i)$ is the indicator function assuming value 1 if ${\hat{y}}_i \le y$ and 0 otherwise. $F_U(y)$ is the cumulative distribution function of a Uniform random variable.

### Index of graphical goodness of the map

We propose to jointly consider the two above-mentioned features in order to build a unique index, where 1/*H* is used to weigh $\sigma _N$. In other words, we propose to select the best map as the one that minimizes the ratio

4 $$\begin{aligned} \phi = \frac{\sigma _{N}}{H} \end{aligned}$$

which is able to take both features 1 and 2 into account. In practice, between two maps with the same spatial variability in the neighborhoods of points, we prefer the one with the highest difference with respect to the Uniform distribution.

It is worth reminding that this index is built with the aim of assessing the graphical appeal, so, as stressed above, it cannot be separated from the simultaneous evaluation of the out-of-sample error. For instance, we have $\phi =0$ in the limiting case of ${\hat{y}}_i$ all equal to the global average, a solution that, in general, has to be discarded due to its extremely limited predictive ability.

All that said, the ratio $\phi$ can be used to tune the algorithm parameters, similarly to what done in Kruppa et al. (2013), where the optimal terminal nodes size is determined by a tuning algorithm designed to offset different characteristics of the algorithm.

**Fig. 11:**
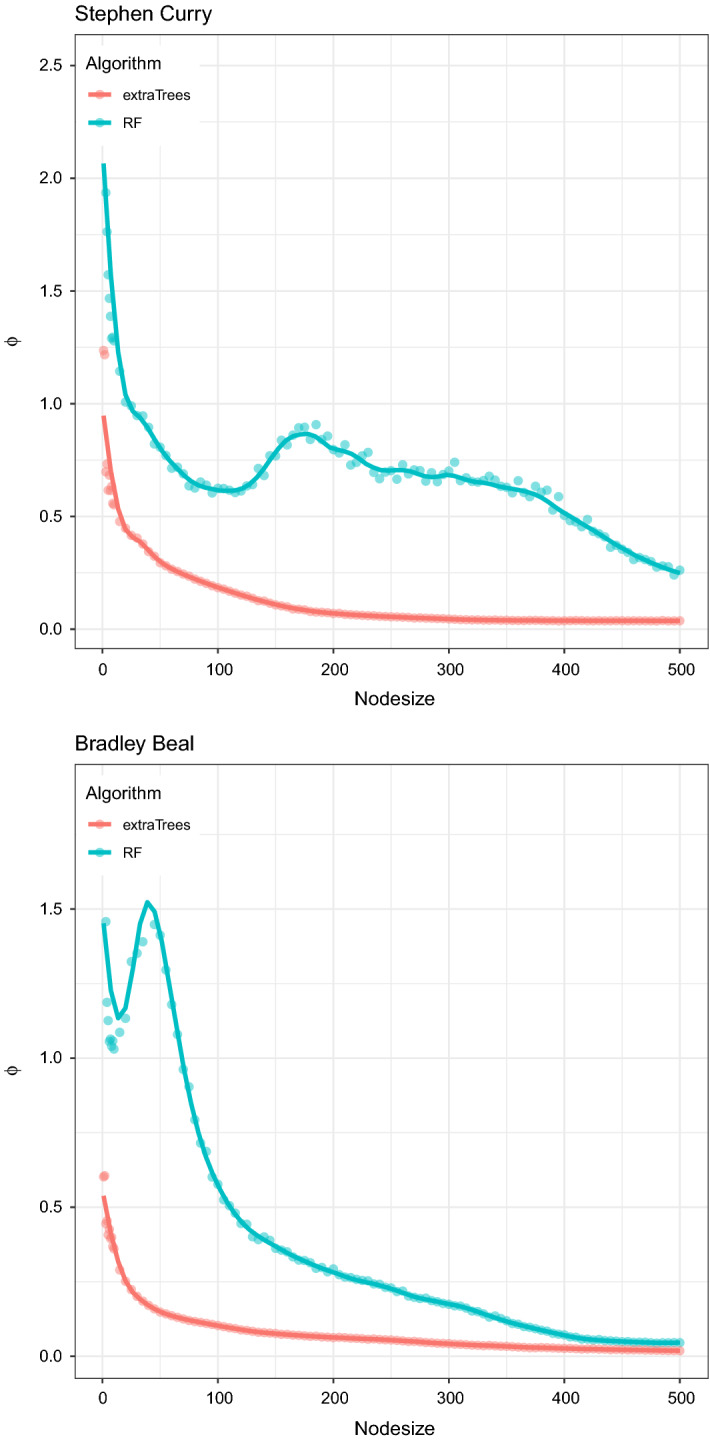
Pattern of the ratio $\phi$ for different values of minimum size of terminal nodes (Nodesize) in ExtraTrees—Stephen Curry (top) and Bradley Beal (bottom), NBA regular season 2020/2021

In Fig. 11 we show the pattern of the ratio $\phi$ for different values of the minimum size of terminal nodes in RF and ExtraTrees. The curves show a substantially decreasing pattern, somehow unsteady for RF. The first clear evidence is that, from the point of view of the graphical goodness, ExtraTrees largely outperform RF, which correspond to the visual perception we had when we considered this issue “by eye”. In addition, the more regular pattern generated by ExtraTrees represents an additional reason to prefer this algorithm.

Limiting attention to ExtraTrees, we can now use the graphs of Fig. 11 in order to select the best value for the minimum size of terminal nodes. In general, we should choose the lowest value that guarantees a sufficiently low value of $\phi$, also considering the pattern of the out-of-sample error shown in Fig. 10, where a node size lower than 80/100 seemed not adequate from the point of view of the generalization error. With respect to these remarks, we think that a good choice is to set the node size equal to 200 for both Curry and Beal, which is just the choice made in the case studies presented in Sect. 4. This provides evidence of the potential usefulness of the ratio $\phi$ as tuning algorithm, when considered jointly with an out-of-sample error measure.

## Concluding remarks

In this paper we have developed the basic idea proposed in Zuccolotto et al. (2021) to analyse basketball players’ or teams’ spatial shooting performance. The aim of the proposed techniques is to produce spatial performance maps able to inform on the scoring probabilities along different areas of the court, with the purpose of delivering—to players and teams—a tool both accurate and easy and immediate to interpret. The first development we have proposed is relative to the use of polar coordinates, which—being expressed in terms of angle and distance with respect to the basket—allow to draw maps more consistent with the court geometry. The second improvement consists in using algorithmic modeling techniques in order to obtain the scoring probabilities’ estimates.

The result is a graphical tool completely new in the basketball analytics literature, able to represent the spatial pattern of the scoring probability with an easy-to-interpret colored basketball court, obtained with procedures that, from a statistical point of view, are innovative, reliable and robust.

In detail, we started by showing the potentialities of using CART, as proposed in the original contribution of Zuccolotto et al. (2021), but we have also produced evidence of some serious shortcomings, mainly related to instability of results as a consequence of small variations in the algorithm’s parameters or in the training data. For this reason we proposed to resort to CART-based ensemble learning algorithms whose structure should overcome the weaknesses of CART, while preserving their strengths.

Finally, from a methodological point of view, we proposed an index to assess the graphical goodness of the spatial performance map, which can be effectively used, jointly with out-of-sample error measures, to determine the optimal values of tuning parameters and to identify the algorithm that should be preferred.

The presented case studies show excellent results from the point of view of both the ease of interpretation of the resulting maps and their robustness. Between the two proposed algorithms, namely Random Forest and Extremely Randomized Trees, the latter seems to be the best performing one.

The proposed graphical tool could be improved by the addition of a categorical covariate (or a numerical one, divided into classes) that is considered to significantly affect the spatial pattern of the scoring probability. Categorical variables can be available in the data set (for example, the variable indicating whether the game is played home or away) or obtained by previous analyses summarizing several other variables, for example by means of cluster analysis, tree-based models, or latent class analysis. In this way, a map would be produced for each category of the covariate. For example, if a player is known to improve his performance from the long distance in the last minutes of the game, a covariate opportunely dividing the game in two or more periods could be efficiently used as predictor.

It is worth nothing that the proposed method can potentially be used to estimate the conditional probability distribution on the court of a dichotomous variable *Y* different from made/missed shot, thus focusing attention on other events of interest occurring during the game.

## Data Availability

Data used in this paper have been made available by BigDataBall (www.bigdataball.com): a data provider that leverages computer vision technologies to enrich and extend sports data sets with a number of unique metrics. Since its establishment, BigDataBall has supported many academic studies as a reliable source of validated and verified statistics for the NBA, Major League Baseball, the National Football League and the Women’s National Basketball Association.

## Footnotes

1 bdsports.unibs.it/basketballanalyzer.

2 The value *n* depends on the grid used to draw the map: in our case we used a $100 \times 100$ grid, so $n=10000$.

## Code Availability

The R codes are available upon request.
